# Transparent Exopolymer Particles in Drinking Water Treatment—A Brief Review

**DOI:** 10.3390/ijerph182312344

**Published:** 2021-11-24

**Authors:** Jianchao Shi, Yongrui Yang, Qitao Yi, Jin Zhang, Lianxiang Wang

**Affiliations:** School of Civil Engineering, Yantai University, Yantai 264005, China; yangyongrui2020@163.com (Y.Y.); yiqitao@163.com (Q.Y.); zhangjin411@outlook.com (J.Z.); wlx17861100653@163.com (L.W.)

**Keywords:** transparent exopolymer particles, algal organic matter, drinking water treatment, membrane fouling, source water reservoir

## Abstract

Transparent exopolymer particles (TEP) have been described as a class of particulate acidic polysaccharides, which are commonly found in various surface waters. Due to their unique physicochemical characteristics, they have recently been receiving increasing attention on their effects in water treatment. Currently, TEP are commonly known as clear, gel-like polysaccharides. This review first introduced the definition of TEP in water treatment and the relationship between TEP and algal organic matter (AOM). Further, in the review, the authors attempt to offer a holistic view and critical analysis concerning the research on TEPs in source water reservoirs, water plants and membrane treatment processes. It was clearly demonstrated in this review that the formation of TEP in source water reservoirs is largely related to water quality and phytoplankton, and the seasonal water stratification may indirectly affect the formation of TEP. In the waterworks, the relationship between TEP and water treatment process is mutual and there is limited research on this relationship. Finally, the mechanism of TEP-induced membrane fouling and the effect of alleviating TEP-induced membrane fouling is discussed in this review. The TEP removed by ultrafiltration can be recombined after membrane, and the recombination mechanism may be an important way to reduce reverse osmosis membrane contamination.

## 1. Introduction

With the development of the economy and increasing industrialisation, algae bloom induced by eutrophication and algal organic matter (AOM) have become a major source of organic pollutants in reservoirs [1]. Hydrophilic organic matter accounts for a substantial part of AOM compared to exogenous natural organic matter (NOM) [2]. The traditional water treatment technique is ineffective in removing hydrophilic and small molecular organics [3]. As a result, AOM causes a series of problems in the treatment of drinking water, such as membrane contamination, which poses a major danger to the water quality safety of urban drinking water [4,5]. By 2030, achieving universal and equitable access to safe and affordable drinking water for all is one of the 17 Sustainable Development Goals (SDGs) of the United Nations [6]. To address these existing challenges, such as AOM in drinking water, it is imperative to rapidly change the economic, engineering and regulatory frameworks that have guided water management policy and investment in the past, and to develop new solutions for sustainable water conservation and development through creativity, research and innovation to ensure scientific and equitable use of water [7].

TEP are a special type of AOM with high viscosity. TEP have been commonly found in surface water, seawater and wastewater and have been described as a class of particulate acidic polysaccharides, which are large, transparent organic particles that can be stained with Alcian blue [8]. TEPs have been extensively analysed in marine ecology, but their significance in water treatment was not realised until 2005 [9,10], as evidenced by the following aspects. Firstly, it has traditionally been considered that the transformation of dissolved organic matter (DOM) into particulate organic matter (POM) is inseparable from the metabolism of microorganisms. Nevertheless, investigations have proven that TEPs can be produced by the spontaneous condensation of DOM under abiotic forces. TEPs provide an abiotic pathway for the transformation of DOM to POM and connect the dissolved substance and granular substance pool in water [11]. Secondly, TEPs play a crucial role in the early stage of biofilm formation. The biofilm that first appeared on a solid surface (such as membrane surface and filler surface) was obtained from TEPs in water rather than from the extracellular polymer (EPS) secreted by microorganisms linked to the solid surface [12]. Finally, TEPs are characterised by high viscosity and easy deformation. It is simple to produce a gel layer on the surface of the membrane or hinder the pore of the filter membrane [13,14]. As a result, TEP is regarded as one of the primary causes of irreversible membrane fouling. TEP-related research is essential for obtaining a comprehensive understanding of AOM and provides a novel perspective for solving the drinking water treatment crisis induced by AOM.

Although the relevance of TEPs as a special form of AOM has been recognised in existing studies, there is still a lack of in-depth research on the development, migration and transformation of TEPs in freshwater systems, particularly in drinking water treatment, resulting in TEP not being taken into account in the design and operation of water treatment. It is critical to systematically study TEPs in the field of drinking water treatment, and there is an urgent need to provide a thorough and systematic review of the functions of TEPs in drinking water treatment. The development, definition, formation and determination of TEP in recent years has been reviewed by Meng et al. [13]; therefore, this study aims to offer an holistic view and critical analysis concerning the research on TEPs in source water reservoirs, water plants and membrane treatment processes.

## 2. Definition of TEP in Water Treatment

Traditionally, the definition of TEP is derived from its determination method. The discovery of TEP in the surface water can be traced back to the 1980s [15], while the quantified of TEP was firstly achieved by Alldredge et al. when they stained the seawater samples with Alcian blue which is specific for negatively charged polysaccharides in 1993 [8]. Then, TEP were operationally defined as particles retained by 0.4 mm tracketched polycarbonate filters and stainable with Alcian blue [16]. According to this similar criterion, Villacorte et al. further defined colloidal transparent exopolymer particles (cTEP, <0.40 mm) and particulate transparent exopolymer particles (pTEP, >0.40 mm) [17].

**Figure 1:**
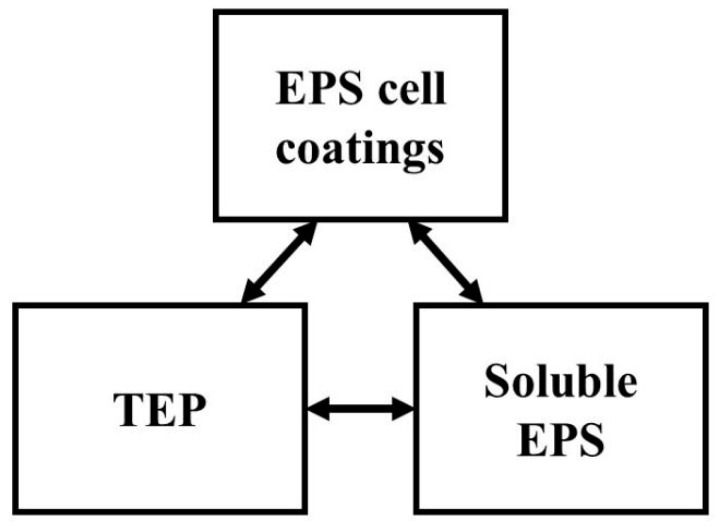
Dynamic relationship between the three pools of EPS. Arrows indicate possible conversions between the pools. The figure is from Thornton (2001) [18].

However, scientists in different fields define TEP slightly differently. Thornton defined TEP as a special form of extracellular polymeric substances (EPS) in his work with diatoms [18]. EPS form three pools in the environment: cell coatings, soluble EPS and TEP. The three different EPS pools are thought to be interchangeable; the dynamic relationship between the three pools of EPS is shown in Figure 1. Generally speaking, the formation of TEP in aquatic environments has two pathways, biotic and abiotic [19,20]. The relationship between TEP and the other two pools of EPS just expresses the biotic pathway. DOM in water can spontaneously aggregate and form POM under the action of shear force of water flow, bubble adsorption and other mechanical forces, and TEP is an important intermediate product of this process.

With the development of detection methods and the in-depth understanding of TEP, TEP are commonly known as clear, gel-like polysaccharides, especially in the area of water treatment [13]. Alcian blue staining is a basic method to determine TEP in water samples. The method proposed by Passow and Alldredge and its updates is still the most used method to determine TEP [16,17,21]. Although this method still defines TEP in terms of their size, the specific chemical properties of TEP are more valuable to study than their size. As is illustrated by Passow (2002), in either of the two pathways, the established TEP and AOM are closely related and have similar properties [3]. More significantly, TEP represents AOM, which is more likely to cause membrane contamination. Although we have many ways to characterize AOM in the laboratory, it is not possible to separate AOM from organic matter and other sources in natural water. Hence, TEP can be used as an important characterization method to study AOM in natural water.

## 3. TEP in Source Water Reservoir

Although there is considerable literature on the distribution, composition and significance of TEPs in natural water ecosystems, few of them study TEPs in water source reservoirs. TEP concentration in water is frequently linked to water type, water quality, phytoplankton and other factors. Table 1 lists the concentration of TEPs in various water sources.

The TEP concentration in lakes and reservoirs ranges from 10 to 10^4^ μg Xeq·L^−1^. For example, the TEP mass concentration in Taihu Lake in China may reach 5 × 10^3^ μg Xeq·L^−1^ [24], while the highest TEP mass concentration in the Mediterranean lake group of Spain may reach 9 × 10^3^ μg Xeq·L^−1^ [26]. In addition, Villacorte et al. discovered that the colloidal state TEPs with the size is <0.4 µm accounts for 64% of the total TEPs, whereas the granular state TEPs with the size >0.4 µm only account for 36% [30]. This reveals that TEPs in water primarily exist in a colloidal state, and their impact on water treatment cannot be neglected.

The spatio-temporal heterogeneity of TEP dispersion in water is evident [31]. Field monitoring indicates that the highest value of TEP concentration often occurs during phytoplankton outbreaks [25]. TEP produced in different algae development periods has variable morphological, physical and chemical characteristics, and the concentration of TEPs in upper and lower water bodies is significantly associated with the phytoplankton density [23]. This demonstrates that studying TEPs in source water is useful in determining the best pretreatment technology to decrease the membrane contamination caused by TEPs in the subsequent process.

However, most study on the concentration and dispersion of TEPs focuses on seawater systems, whereas the related reports in freshwater are relatively few. Furthermore, reservoirs, as drinking water sources, often have large water depths, and their water stratification phenomenon has a significant effect on water quality over a period [32,33]. It is still unknown how water thermal stratification affects the concentration, distribution, physical and chemical properties of TEPs.

Meanwhile, because TEP is a gel, the size structure of TEP in water is impacted by the reaction time and the environmental pH value according to the polymer gel theory [34]. The production, reproduction and metabolism of algae are profoundly affected by the stratified structure of water reservoir with increasing depth and the seasonal variation of water quality, and the change in water body pH value, flow velocity is also related to water body stratification [35,36]. This indicates that seasonal stratification of the water body can influence TEP formation directly by affecting the phytoplankton and pH value of the water body.

## 4. TEPs in Water Treatment Process

### 4.1. Removal of TEP by Water Treatment Process

The essence of TEPs is an acid polysaccharide. Studies have revealed that it has a good relationship with COD and TOC. As a result, the technique that can eliminate COD and TOC can also remove TEP [28]. Table 2 show the removal of TEP by different water treatment processes reported in literature. The removal of TEP by different membrane processes reported in literature is shown in Table 3.

According to studies conducted by Nevel et al. in two water plants in Belgium, the combination of coagulation and sand filtration can efficiently decrease TEP content, with a removal rate of 67%, UF to TEP removal rate of 9%, whereas the RO process can remove all TEPs with a removal rate of 100% [28]. Villacorte et al. have similar conclusions, that is, neither conventional water treatment process nor UF technique can eliminate TEPs from water, particularly colloidal TEPs, whereas RO can eliminate TEPs [30,39]. Li et al. evaluated the coagulation properties of TEPs and discovered that using ferric chloride as a coagulant and controlling pH can convert the colloidal TEP state into the granular TEP state, thereby decreasing the load on the membrane filtration, and increasing the dosage of the coagulant is useful to enhance the removal rate of TEP [40].

Coagulation, on the other hand, cannot be treated as a separate process and must be complemented with advanced treatment procedures, such as sand filter and UF. Furthermore, several studies evaluated the removal impact of biofilter, membrane bioreactor, bioactive carbon and other methods on TEPs [37,38,41,42].

### 4.2. Role of TEP in the Water Treatment Process

In the process of drinking water treatment, TEP, as an organic pollutant, should be removed first. Meanwhile, TEP has a significant influence on the water treatment process due to its special chemical properties. The aforementioned studies focus mostly on the impact of the TEP removal process, neglecting to consider TEPs’ potential influence on the water treatment unit process as an organic matter with high viscosity, low density, easy deformation and negative charge.

For the coagulation process, the high viscosity of TEPs binds inorganic particles and NOM like bridges and promotes coagulation, but the carboxylic acid groups R-COO^−^ and sulfonic acid groups R-SO_3_^−^ in TEPs reduces the coagulation effect and the increasing of coagulant dosage [11]. For example, Bar-Zeev et al. studied biological flocculation in a novel, large pilot-scale, two-stage granular rapid bioflocculation filter (RBF). Their study found that, over a year-long study, the pilot RBF and the conventional rapid sand filtration showed similar filtration efficiencies. TEP produced by microorganisms in RBF showed melancholy flocculation properties and if it can be used properly, the flocculation process does not require chemical additives [43,44].

The solid–liquid separation ability of destabilised floc is crucial for removing the impurities in water. Impurities in water, on the other hand, will have a decisive effect on the solid–liquid separation capacity of flocs. The quality of destabilised flocs will inevitably be influenced by the special disparity between NOM and AOM. If any research finds that with the increase of water flow shear force, the strength of flocs generated by AOM gradually diminishes, which is exactly the opposite of the performance of NOM [3]. Therefore, it is essential to analyse the influence of TEPs, as a special form of AOM, influence on the coagulation and solid–liquid separation processes.

### 4.3. TEP in Drinking Water

TEP is widely found in many types of water, including seawater, surface water, groundwater and sewage [11,45]. However, whether TEP exists in drinking water is rarely reported. When studying the TEP removal in different water plants, Nevel et al. found that few or no TEP could be detected in the final drinking waters at time of sampling [28]. This is the only article that directly describes TEP concentration in drinking water in the existing literature, so it has important research significance.

However, compared with the similar results of other studies, it is found that there are several contradictions that need further study and explanation. The first, plant A studied by Nevel et al., after RO treatment, TEP concentration slightly increased when the water entered the remineralization pond. However, it is not clearly explained how the newly formed TEP is removed by the subsequent process, nor is it clear whether TEP will be formed again. Second, deformation and reorganization is an important feature of TEP; this has been confirmed by research [30]. However, the conditions for TEP recombination in filtered water, such as flow rate, shear force and organic matter level, are still unknown. In other words, the distribution characteristics of TEP in the water supply network are worth further study. Moreover, TEP has been confirmed to play an important role in the early stage of biofilm formation, which is closely related to the biological stability of water.

As a special form of AOM, TEP is a precursor of disinfection byproduct just like AOM. Even though disinfection byproducts in drinking water are potentially carcinogenic to human health, TEP plays an important role in water treatment in membrane fouling and biofilm formation.

## 5. Membrane Contamination Caused by TEPs

### 5.1. Mechanism of TEP-Induced Membrane Fouling

**Figure 2:**
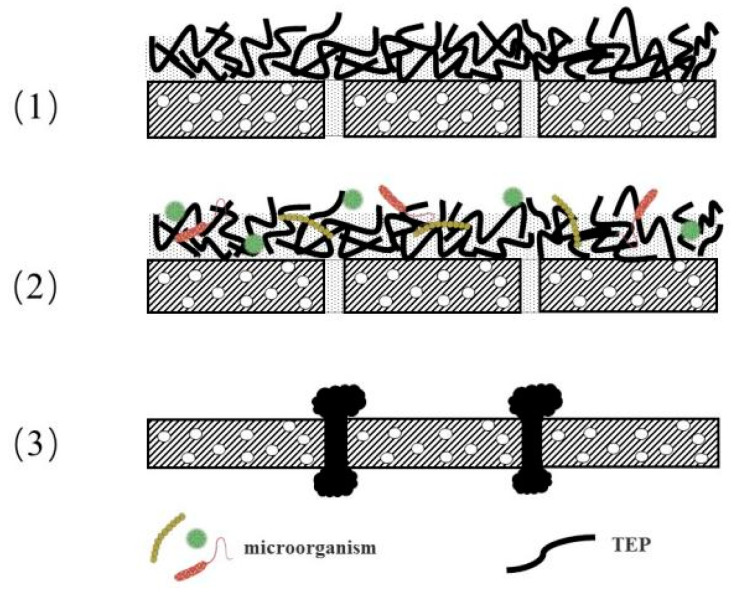
Membrane fouling mechanisms by TEP described in the literature. Information is from Villacorte (2013) [47] and Meng (2013, 2018, 2019) [46,48,49].

TEP is an important substance for membrane fouling due to its high molecular weight and high viscosity [46]. As illustrated in Figure 2, there are three primary factors that contribute to membrane fouling.

Firstly, TEPs’ viscosity is 2–4 times higher than that of most organic substances, and they are easily adsorbed on the surface of various biological or non-biological solids. Further, a highly crosslinked three-dimensional network structure gel layer is produced, thus decreasing the membrane flux. Secondly, TEPs’ three-dimensional network structure provides a habitat for microorganisms and has become a carrier of microorganisms to survive on the membrane surface. Over time, serious biological-organic composite membrane fouling occurs. Thirdly, TEPs are easily deformed, thus can pass through the membrane pores with a size smaller than their size, and some TEPs get stuck in the membrane pores, thus blocking the pores [47,48,49,50].

In the membrane separation process, TEPs with varied particle sizes, structures and qualities exhibit distinct membrane fouling mechanisms. Through intermolecular crosslinking, Meng et al. discovered that sodium alginate with few molecules generates TEPs with a simple structure and small size [48]. The microfiltration membrane fouling conforms to the membrane pore blocking mechanism, whereas xanthan gum molecules are moderately large. The TEPs generated by the combination have a complicated network structure, and their microfiltration is more inclined to the filtration mechanism of the filter cake layer. Zhang et al. established that the free type TEP has a small particle size and is easy to block membrane pores during UF, causing serious irreversible membrane fouling, whereas the fixed type TEP fixed on algae cells is simple to produce a filter cake layer on the membrane surface to induce reversible fouling, owing to the large particle size of algae cells [51]. As a result, the structure and force of the adsorption layer on the membrane surface of TEP can be properly described, which is critical for understanding the membrane fouling change mechanism of TEP. The dissipative quartz crystal microbalance QCM-D, for example, can monitor the adsorption amount of TEP on the membrane and the adsorption layer in real-time with high sensitivity, and the atomic force microscope can directly measure the interface microscopic force linking the membrane [52,53].

In addition, researchers such as Villacorte also discovered that in the UF-RO water treatment system, the TEPs eliminated by UF were partially rapidly reborn and eventually deposited on the surface of the RO membrane, indicating that there are still some TEP precursors in the UF effluent and the regeneration of TEPs occur in the pipeline [30,54]. The development of TEPs is related to cations in water. Cations in water influence the crosslinking degree of TEP precursors, like polysaccharides, through intermolecular forces. The mixture of monovalent, divalent and trivalent cations with polysaccharides is connected to the functional groups and their spatial configuration [55,56]. According to Passow, the water flow shear force may stretch and straighten out a single polymer fibre, thus promoting the organisation of these polymers to form TEPs, indicating that the water flow shear force has an essential influence on the secondary formation of TEPs [11,20].

### 5.2. Effect of Alleviating TEP-induced Membrane Fouling

TEP-induced membrane fouling was first found in seawater RO. Many seawater desalination plants take TEP as an important indicator to evaluate the potential of membrane pollution because TEP differs from other forms of EPS such that due to their particulate structure, they can aggregate and also be collected by filtration, whereas dissolved EPS cannot [57].

To reduce TEP-induced membrane fouling, pretreatment is frequently considered the Achilles heel of an RO. The selection of pretreatment process is greatly affected by the quality of raw water, and the water pretreatment process is not a single method but a combination of many methods. Different pretreatment methods and the length of pretreatment process have certain influences on the total investment of the project [58]. A breakdown of average capital expenses for seawater RO facilities indicates that the pretreatment cost accounts for 12% of the total investment [59]. Therefore, under the same conditions, choosing the right process can effectively save costs. The investment estimates of common combined pretreatment processes are shown in Table 4.

As is shown in Table 4, common pretreatment methods include coagulation, sedimentation, flotation, filtration and UF. UF for pretreatment is not only technically feasible, but also economical. UF replaces the multi-media filtration process, the subsequent RO can produce 20% more water than the conventional method because of the better water quality, and RO membrane has a long running time and low running cost. Therefore, compared with conventional methods, UF has a low price [60]. However, the latest research shows that TEP can also cause UF membrane fouling too [47,55]. It is suggested that the formation of TEP in membrane process influent should be reduced by adjusting the chemical properties of water. For example, adding electrolytes (such as Ca^2+^) can reduce the negative charge on the surface of cTEP and TEP precursors, reduce the electrostatic repulsion force, and promote cTEP to form pTEP, which helps to reduce the resistance of UF membrane [29,56]. Lin et al. also reached a similar conclusion when they treated boron and TEP in seawater synchronously with FeCl_3_ [61].

## 6. Conclusions and Prospects

TEP are commonly known as clear, gel-like polysaccharides have been commonly found in feed water and in different water treatment processes. The main implications of this review and the research prospects of TEP are as follows:

(1) It is difficult to separate AOM from organic matter and other sources in natural water. TEP can be used as an important characterization method to study AOM in natural water due to special chemical properties in TEP.
(2) The seasonal water stratification has a significant impact on water quality and phytoplankton reproduction and may indirectly affect the formation of TEP in water. However, there is a lack of systematic investigation and research on the formation and the temporal and spatial distribution of TEP in a source water reservoir with a certain depth of water where stratification may occur.
(3) The relationship between TEP and water treatment process is mutual. The impact of TEPs on the conventional water treatment process is an urgent problem to be addressed.
(4) Mechanism of TEP-induced membrane fouling can be explained from three aspects: the formation of cake layer, provision of nutrients for microorganisms and a plug membrane channel. In addition to improving membrane materials, it is also a feasible way to reduce the generation probability of TEP by regulating the inlet water quality.

## Figures and Tables

**Table 1:** Overview of TEP concentrations in fresh water.

Sample Type	pTEP	cTEP	Reference
	μg Xeq·L^−1^	μg Xeq·L^−1^	
Neuse River Estuary (Jan Apr), USA	991~1712	/	[22]
Neuse River Estuary (May–Aug), USA	805~1801	/	[22]
Neuse River Estuary (Aug), USA	>3500	/	[22]
Pearl River Estuary (Jan), China	88.7~1586.9	/	[23]
Pearl River Estuary (Aug), China	521.5~1727.4	/	[23]
Lake Taihu, China	0~5190	/	[24]
Lake Kinneret, Israel	759~2385	/	[25]
Mediterranean lakes, Spain	66~9038	/	[26]
North temperate lakes, USA	36~1462	/	[26]
Quentar Reservoir, Spain	1.9~335.2	/	[27]
Surface water, Belgium	14.8 ± 14	684 ± 94	[28]
Ground water, Belgium	<5	<50	[28]
Secondary wastewater effluent, Belgium	102 ± 20	1470 ± 189	[28]
Surface water in a pond, Belgium	2~143	5~137	[29]
Meuse River (Jul), The Netherlands	~105	~165	[30]
Lake IJssel (Jun), The Netherlands	~110	~500	[30]
Gent-Terneuzen canal (Jul), Belgium	~80	~330	[30]
River Estuary (Jul), Belgium	~230	~290	[30]
Surface water, The Netherlands	990	/	[31]

“/” means there is no data available.

**Table 2:** The removal of TEP by different water treatment processes reported in literature.

Water Treatment Processes	Feed Water	Key Description	Reference
Prechlorination	Secondary wastewater effluent	Increased cTEP and pTEP concentrations with respectively 34 and 41%	[28]
Coagulation+Sedimentation	Surface water	A decrease of cTEP amount and an increase of pTEP weight, while total TEP concentrations did not change significantly	[28]
Coagulation+Flotation	Surface water	The pTEP amount stayed minimal and the cTEP concentration decreased by 70%	[28]
	River water	Decreased the total TEP concentration further with 70%	[17]
Filtration	Effluent after Coagulation+Sedimentation	A good option to remove these coagulated pTEP (decrease ~90%) but was a too rough method to abate the smaller cTEP (decrease ~5%)	[28]
	Effluent after Coagulation+Flotation	The pTEP amount stayed minimal and the cTEP concentration increased	[28]
	In-line coagulation	The removal of TEP was 70% while the remaining fraction of TEP was totally removed by UF	[31]
	Coagulation Effluent	TEP concentrations in the input seawater were diminished by 27% (±19) after passing the stage of the sand/ mixed-bed filter	[37]
Activated carbon	Filter Effluent	Decreased the cTEP concentration further with 50%	[28]
Biological activated carbon filter	Seawater	The AOC and TEP concentration in seawater was reduced significantly by 90% and 84%, respectively	[38]

**Table 3:** The removal of TEP by different membrane processes reported in literature.

Membrane Processes	Feed Water	Rejection Rates	Reference
Microfiltration (MF)	Canal water	0% pTEP, cTEP ~70%	[30]
	Estuary water	~65% pTEP, ~50% cTEP	[30]
	Surface water	95% pTEP, 97% cTEP ^※^	[28]
Ultrafiltration (UF)	Surface water	100% pTEP, 17~67% cTEP	[30]
	coagulation effluent	100% pTEP	[31]
	Filtration effluent	95% pTEP, 97% cTEP ^※^	[28]
	coagulation effluent	~100% pTEP, ~99% cTEP	[37]
	coagulation effluent	26~29% total TEP	[38]
Reverse osmosis (RO)	UF effluent	100%	[30]
	Surface water	100%	[28]

^※^ UF membranes with a pore size as big as 100 nm.

**Table 4:** The investment estimates of common combined pretreatment processes. Information is from Wu et al. (2021).

Pretreatment Processes	Investment (Million ¥)	Notes
Micro-Flocculation + Multi-media Filtration	7.0~10.0	
Coagulation + Sedimentation + Filtration + UF	10.0~13.0	Includes sludge treatment systems
Micro-Flocculation + Filtration + UF	11.0~13.0	
Coagulation + Flotation + Filtration + UF	10.0~14.0	Includes sludge treatment systems

## Data Availability

The datasets generated and/or analysed during the current study are available from the corresponding author on reasonable request.

## Abbreviations

The following abbreviations are used in this manuscript:

TEP	Transparent exopolymer particles
cTEP	Colloidal transparent exopolymer particles
pTEP	Particulate transparent exopolymer particles
AOM	Algal organic matter
NOM	Natural organic matter
DOM	Dissolved organic matter
POM	Particulate organic matter
EPS	Extracellular polymer
COD	Chemical oxygen demand
TOC	Total organic carbon
RO	Reverse osmosis
UF	Ultrafiltration
MF	Microfiltration
